# [18F]Flortaucipir distinguishes Alzheimer’s disease from progressive supranuclear palsy pathology in a mixed-pathology case

**DOI:** 10.1007/s00401-019-02121-w

**Published:** 2020-01-04

**Authors:** Ruben Smith, Daria Pawlik, Christer F. Nilsson, Elisabet Englund, Oskar Hansson

**Affiliations:** 1 Department of Neurology, Skåne University Hospital, Lund, 20502 Malmö, Sweden; 2 Clinical Memory Research Unit, Department of Clinical Sciences Malmö, Lund University, Lund, Sweden; 3 Division of Oncology and Pathology, Department of Clinical Sciences, Lund University, Lund, Sweden; 4 Memory Clinic, Skåne University Hospital, Malmö, Sweden

Radiotracers for AD-like tau aggregates have recently become available for positron emission tomography (PET) studies in neurodegenerative disorders. We have shown that [^18^F]Flortaucipir binds to tau pathology in Alzheimer’s disease (AD) [5]. Using autoradiography in postmortem tissue no convincing binding of [^18^F]Flortaucipir to tau pathology has been found in progressive supranuclear palsy (PSP) [1, 3]. However, we and others have shown increased in vivo retention of [^18^F]Flortaucipir in the basal ganglia, but not in the cortex, of patients with PSP [2, 3]. Here we present data from a 78-year-old patient, who was clinically diagnosed with progressive non-fluent aphasia (PNFA), after a 3-year period of increasing speech difficulties. When first evaluated by a speech therapist in 2009, the patient presented with problems regarding word repetition and fluency of speech under time pressure. He managed the Boston naming test without problems, but showed difficulties with spontaneous word generation (letter- and animal-fluency tests). MMSE performed in 2012, 3 years into the disease, was 24/30 with difficulties in attention/counting, language repetition and the written sentence was grammatically incorrect. Orientation, memory and visuospatial abilities were preserved. Answers were limited to single words with addition of repetitive nonsense syllables at the end of the words. Letter- and animal-fluency tests could no longer be performed due to limited spontaneous speech. Cerebrospinal NFL levels were increased and β-amyloid decreased, but tau and phospho-tau were within normal limits. It gradually became increasingly difficult to spell and read. Approximately 5 years after the onset of the speech difficulties, the patient could only generate a monotonous repetitive sound. The patient could understand speech and produce written answers to questions. MMSE at this timepoint was 20/30, with deficits in delayed memory recall and orientation being added to the deficits found in 2012. Alongside the speech impairment there was a slow but steady cognitive decline with reduced stress tolerance, irritability and an emotional lability, but apart from a left arm hyperreflexia the patient never exhibited motor symptoms. In 2016, the patient was admitted to a nursing home. He passed away due to bilateral bronchopneumonia in 2017.

Twenty months prior to death, the subject underwent [^18^F]Flortaucipir PET and MRI scanning. [^18^F]Flortaucipir PET showed retention bilaterally not only in the basal ganglia, but also retention in the temporal cortices (left more than right) (Fig. 1a and online Supplementary Fig. 1a). For methods, see Supplement, online resource. Neuropathological assessment postmortem revealed a diagnosis of PSP, with coexisting AD pathology. Amyloid immunohistochemistry showed frequent neuritic plaques (Thal phase 5), along with the tau pathology corresponding to Braak stage V (for details, see online supplement). There was atrophy of the midbrain and a slight general cortical atrophy that was more pronounced in the left temporal lobe, but atrophy was less widespread than [^18^F]Flortaucipir retention (online Supplementary Fig. 1b). Macroscopically there was depigmentation of locus coeruleus and substantia nigra. Microscopy showed AT8-positive tufted astrocytes scattered throughout the cortex and cerebral white matter confirming a diagnosis of PSP. AT8-immunohistochemistry further showed dense taupositive neurites and AD-type neurofibrillary tangles in the temporal lobes bilaterally (more left than right). Immunohistochemistry for 4R and 3R tau inclusions showed positivity for 4R and 3R pathology in the temporal lobes, indicative of AD pathology, but only 4R pathology in the frontal lobe, suggestive of isolated PSP pathology in this region (Fig. 1b). TDP43 and alpha-synuclein immunohistochemistry were negative.

**Fig. 1:**
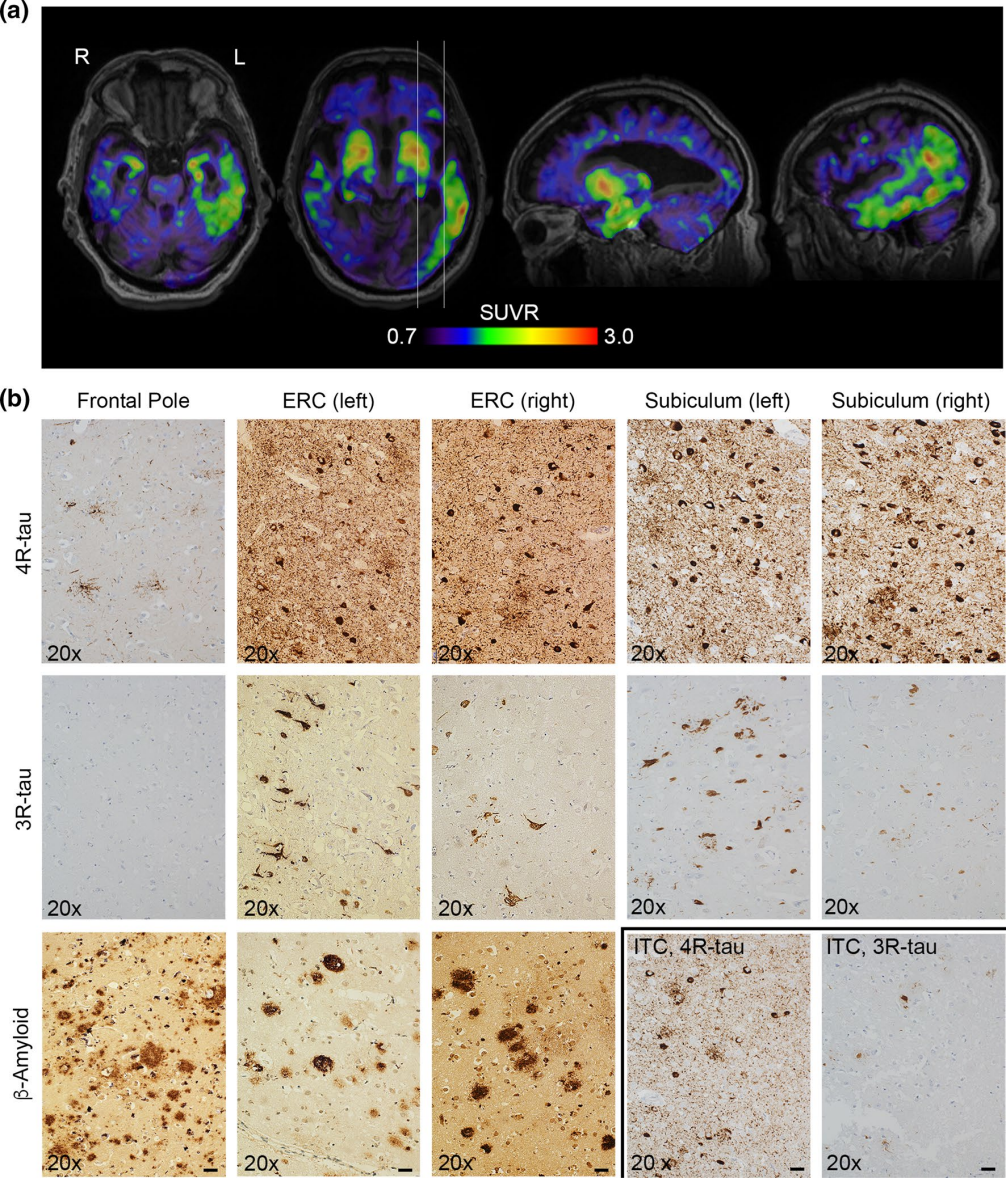
[^18^F]Flortaucipir imaging and neuropathology. **a** [^18^F]Flortaucipir images showing retention primarily restricted to the temporal lobes. The white lines indicate the locations of the sagittal images to the right. *L* left, *R *right, *SUVR *standardized uptake value ratio. **b** Images of 4R tau (upper row and bottom row, 2nd image from the right), 3R tau (middle row and bottom row, rightmost image), and β-amyloid (bottom row, three leftmost images) from frontal pole, right and left entorhinal cortices, right and left subiculum and right inferior temporal cortex (ITC). Hematoxylin and eosin stainings are available in online Supplementary Fig. 2. Scale bars indicate 20 µm

Neuropathology showed relatively sparse PSP-like tau pathology in the basal ganglia (online Supplementary Fig. 2). In the PET scan, we found retention in the basal ganglia, but these are also well-known areas of age-related off-target binding of [18F]Flortaucipir [1, 3]. At a group level, we have reported increased retention in PSP compared to controls [3], but the ranges were overlapping. We have further reported a poor correlation of cortical PSP pathology to [18F]Flortaucipir PET retention in a case with clinical PSP-Richardson syndrome [4] indicating that [18F]Flortaucipir has a lower affinity for the 4R tau pathology in PSP compared to the 3R/4R tau pathology of AD. It is also possible that the amount of tau pathology present in the brain of patients with PSP is insufficient to result in a clearly discernible PET signal. Taken together, these results indicate that the basal ganglia retention in PSP is likely mainly explained by off-target binding, with the possible addition of some specific signal derived from PSP-tau pathology.

The rather long timespan of 20 months between imaging and autopsy may be a limitation of the report.

In conclusion, we find [^18^F]Flortaucipir PET retention in cortical areas affected by AD-(3R and 4R)-pathology, but not in areas solely affected by PSP-(4R)-pathology in this PNFA case with mixed AD and PSP pathologies. The reason for the tau tracer not reflecting the tau pathology of PSP may be due to a lower affinity of [^18^F]Flortaucipir for straight 4R-filaments, lower abundance of the 4R tau inclusions, or a combination.

## Electronic supplementary material

Below is the link to the electronic supplementary material.

Supplementary file1 (DOCX 11043 kb)
