# Supplementary material for: [18F]Flortaucipir distinguishes Alzheimer’s disease from progressive supranuclear palsy pathology in a mixed-pathology case
Source: Acta Neuropathol. 2020 Jan 4;139(2):411–3. doi: 10.1007/s00401-019-02121-w (PMC6989415; doi:10.1007/s00401-019-02121-w)
Supplement: Supplementary file 1 — Supplementary file1 (DOCX 11043 kb) [file 401_2019_2121_MOESM1_ESM.docx]

*Supplementary material*

**[18F]Flortaucipir distinguishes Alzheimer’s Disease from Progressive Supranuclear Palsy pathology in a mixed-pathology case**

Ruben Smith, Daria Pawlik, Christer F Nilsson, Elisabet Englund, and Oskar Hansson.

**Methods**

*Participant*

All procedures conformed to the Declaration of Helsinki and were approved by the Regional Ethical Review Board at Lund University as well as the Swedish Medical Products Agency. The patient described in this case report participated in the Lund Prospective Frontotemporal dementia Study (LUPROFS), a longitudinal study of frontotemporal disorders. Informed written consent was obtained from the patient before inclusion in the study.

For comparative purposes in the voxel-wise analyses 54 cognitively and neurologically healthy controls having undergone [18F]Flortaucipir PET were included. The inclusion criteria of the controls are further detailed in [4].

*MR and PET Imaging*

The subject underwent 3.0T MRI-scans on a Siemens Skyra scanner (Siemens Medical Solutions, Erlangen, Germany). Acquired sequences were fluid-attenuated inversion recovery (FLAIR) and T1-weighted magnetization-prepared rapid gradient echo (t1-mprage).

Synthesis of the radiotracer has been described previously[3]. Emission data was acquired in LIST-mode using a GE Discovery 690 PET/CT (GE Healthcare, Milwaukee, USA), 80-100 min after an intravenous bolus injection of 370 MBq of [^18^F]Flortaucipir. Data was reconstructed as previously described[3]. LIST-mode data were binned into 4x5 min time-frames. PET data was analyzed further in an in-house developed pipeline where the data were motion corrected, summed and co-registered to the t1-mprage MRI and normalized to Montreal Neurological Institute (MNI 152) space. The MRI was skull stripped and segmented into grey and white matter. The MRI was normalized to template space and FreeSurfer 6.0 ROI parcellation was applied to the grey matter. A standardized uptake value ratio (SUVR) image was created using the inferior cerebellar grey matter as a reference region. The image was coregistered to the MRI using Pmod version 3.711 (Pmod technologies llc., Zurich, Switzerland) for visualization purposes (Fig 1, Suppl Fig 2).

Voxel-wise PET comparisons were made using SUVR images in MNI152 space. MRI images were compared using voxel-based morphometry (VBM) in SPM12 (Wellcome Department of Cognitive Neurology, London, UK; http://www.fil.ion.ucl.ac.uk/spm) in MATLAB (v. 9.2, 2017b). In brief, native space T1 images were segmented, DARTEL template was calculated, grey matter volumes normalized to MNI space. The grey matter volumes were compared using a two-sample t-test, adjusting for intracranial volumes. Significant clusters were identified at p (uncorrected) <0.001, and thresholded at cluster size (k) 600, (FDR <0.05). Results are visualized as surface projections on Human PALS-B12 brain templates using Caret v5.65 software [5].

*Neuropathology*

The post mortem interval was 150 hours. The brain was fixed with immersion and perfusion in formaldehyde solution (4 %), cut in bi-hemispheric coronal sections and embedded in paraffin. Neuropathological analysis was performed on coronal sections including the basal ganglia, the hippocampus/the inferior frontal gyrus, the frontal pole and the parietal and occipital cortex, plus sections covering the midbrain and the cerebellum. Paraffin-embedded sections were stained with hematoxylin and eosin (H&E). Immunohistochemistry was performed on 4 μm-thick tissue sections using antibodies for phosphorylated tau (AT8, 1:200, DAKO), 4R tau (Anti-4R-tau, rabbit polyclonal antibody, 1:3000, CosmoBio), 3R tau (RD3, 8E6/C11, 1:1000, Millipore), α-synuclein (clone LB509, 1:600, Zymed laboratories), TDP-43 (pS409/410, clone 11-9, 1:5000, CosmoBio) and β-amyloid (4G8,1:500, BioLegend) . All immunohistochemical sections were microwave pre-treated in 10 mM citrate buffer (pH 6.0) for 10 minutes at 100°C, for antigen retrieval. The staining procedure (DAKO ChemMate Kit Peroxidase/3,3′-diaminobenzidine) was performed using an automated immunostainer (Dako Autostainer Plus, DAKO Sweden AB).

Alzheimer Disease pathology was assessed using ABC-criteria based on the National Institute on Aging–Alzheimer’s Association guidelines for the neuropathologic assessment of Alzheimer’s disease [1, 2]. The Thal phase was five (corresponding to A3), the Braak stage was judged to be V (B3) due to the presence of neurofibrillary tangle tau pathology in the occipital lobe (peristriate cortex). The CERAD score was frequent (C3).

**Supplementary discussion**

*Symptom development and underlying pathology.*

The symptoms of our patient were those of a progressive non-fluent aphasia, with the addition of delayed memory recall problems a couple of years into the disease. Due to the initial isolated language symptoms we believe that the symptoms initially were mainly driven by PSP-pathology, but that our patient gradually developed an incipient AD underlying the problems in memory retention. However, the AD pathology also likely, due to the left temporal lobe localization, accelerated the language difficulties.

**Supplementary Figures**

**Supplementary Figure 1**

**
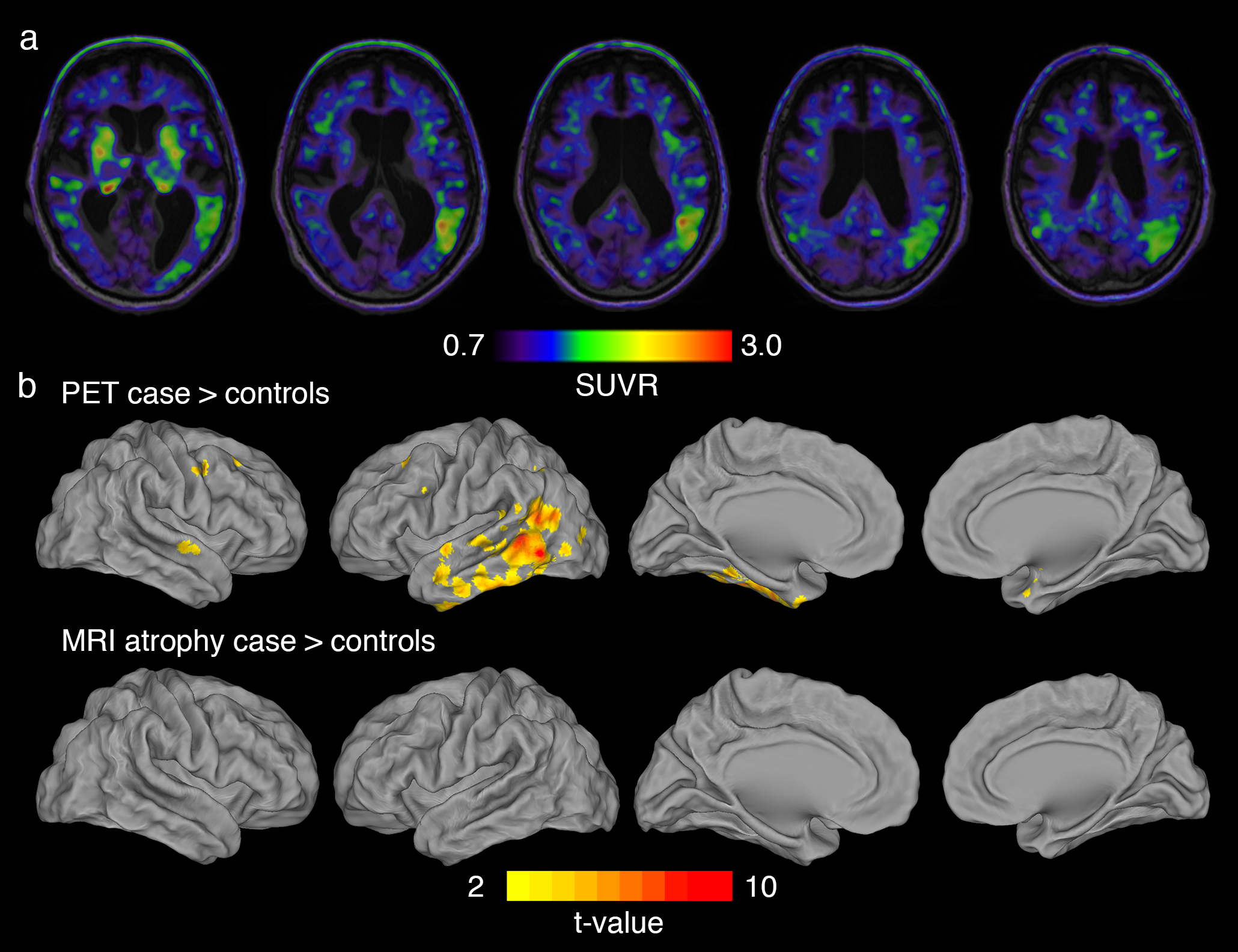
**

Panel a) shows transversal [18F]Flortaucipir SUVR images of the patient overlaid on the MRI. The images are shown in radiological orientation with the left side of the patient depicted to the right. Panel b) shows significant voxels from the voxel-wise comparison to 54 healthy controls for [18F]Flortaucipir PET (upper row) and MRI (bottom row; no significant voxels).

**Supplementary Figure 2**


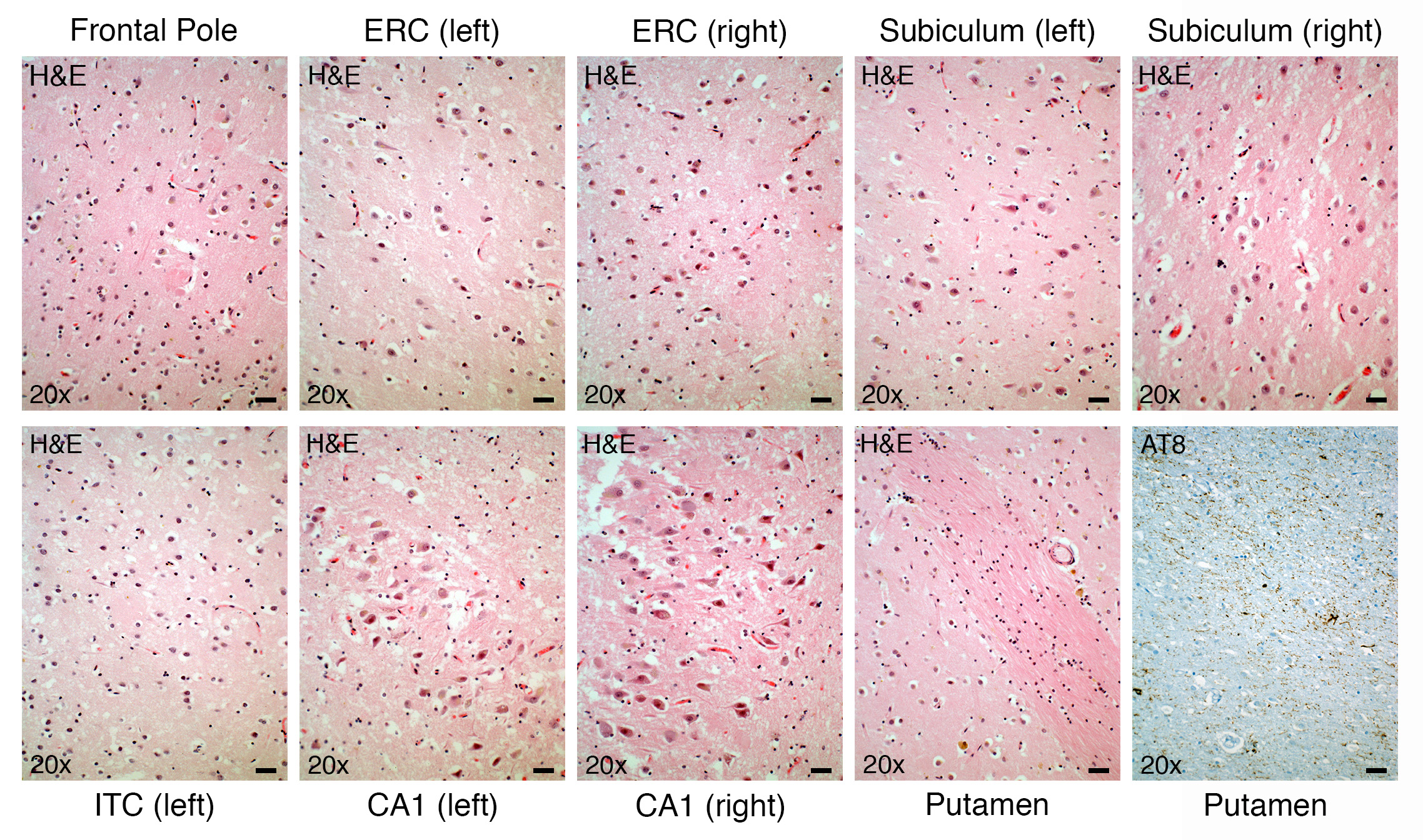


Hematoxylin – Eosin staining of the regions shown in Figure 1, of the CA1 region in the hippocampus, and of the putamen at 20x magnification. Bottom row rightmost image shows AT8 (phospho-tau) immunohistochemistry of the putamen. Scale bars indicate 20 µm.

**Supplementary acknowledgement**

We are grateful to the patient and to the family for participating in the study and to Avid radiopharmaceuticals for providing the precursor of [^18^F]Flortaucipir, we also thank Drs. Jonas Jögi and Tomas Olsson, Skåne University Hospital for PET data acquisition and radiotracer synthesis, and Prof Irina Alafuzoff, Uppsala University for 3R/4R immunohistochemistry. The study was supported by the European and the Swedish Research Councils.

**References**

1 Hyman BT, Phelps CH, Beach TG, Bigio EH, Cairns NJ, Carrillo MC, Dickson DW, Duyckaerts C, Frosch MP, Masliah Eet al (2012) National Institute on Aging-Alzheimer's Association guidelines for the neuropathologic assessment of Alzheimer's disease. Alzheimers Dement 8: 1-13 Doi 10.1016/j.jalz.2011.10.007

2 Montine TJ, Phelps CH, Beach TG, Bigio EH, Cairns NJ, Dickson DW, Duyckaerts C, Frosch MP, Masliah E, Mirra SSet al (2012) National Institute on Aging-Alzheimer's Association guidelines for the neuropathologic assessment of Alzheimer's disease: a practical approach. Acta Neuropathol 123: 1-11 Doi 10.1007/s00401-011-0910-3

3 Smith R, Schain M, Nilsson C, Strandberg O, Olsson T, Hagerstrom D, Jogi J, Borroni E, Scholl M, Honer Met al (2017) Increased basal ganglia binding of 18 F-AV-1451 in patients with progressive supranuclear palsy. Mov Disord 32: 108-114 Doi 10.1002/mds.26813

4 Smith R, Scholl M, Leuzy A, Jogi J, Ohlsson T, Strandberg O, Hansson O (2019) Head-to-head comparison of tau positron emission tomography tracers [(18)F]flortaucipir and [(18)F]RO948. Eur J Nucl Med Mol Imaging: Doi 10.1007/s00259-019-04496-0

5 Van Essen DC, Drury HA, Dickson J, Harwell J, Hanlon D, Anderson CH (2001) An integrated software suite for surface-based analyses of cerebral cortex. J Am Med Inform Assoc 8: 443-459
